# Unfair differentiation, fragmented interventions, and gender reinforcement: a qualitative study of how students experience teachers’ school climate work on gender

**DOI:** 10.3389/fpsyg.2026.1659564

**Published:** 2026-03-03

**Authors:** Camilla Forsberg, Robert Thornberg, Eva Hammar Chiriac

**Affiliations:** Department of Behavioural Sciences and Learning, Linköping University, Linköping, Sweden

**Keywords:** gender, qualitative analysis, school climate, socio-ecologic framework, student-teacher interactions

## Abstract

Characteristics of the school climate are crucial because they can influence students’ academic achievement, wellbeing, and interpersonal relationships, including peer aggression. Teachers play a significant role in promoting a positive school climate grounded in care, fairness, support, and responsiveness. However, qualitative research on how students experience and reflect upon their school climate remains limited. In this qualitative study, we explored students’ perspectives on student-teacher relationships, school climate, and gender. Fifty-nine focus group interviews were conducted with students in grades 1–9 (ages 7–15 years) from two schools in Sweden. The data were analyzed using constructivist grounded theory. The findings revealed that students experienced a gender-normative teacher practice in everyday student-teacher interactions and in teachers’ broader efforts to shape the school climate. This included gender reinforcement, fragmented interventions in student-to-student incidents, and unfair differentiation in classroom management practices. From the students’ perspective, the qualitative findings illustrate how taken-for-granted gender norms may at times influence teachers to engage in interactions and practices that constrain gender identities, reinforce inequality, and potentially undermine a supportive school climate.

## 1 Introduction

A well-established connection exists between the characteristics of the school climate and students’ academic achievement, wellbeing, and interpersonal relationships, including peer aggression (Aldridge and McChesney, 2018; Aldridge et al., 2018; Demirtas-Zorbaz et al., 2021; Kutsyuruba et al., 2015; Zych et al., 2019). School climate is a multidimensional construct encompassing to “shared beliefs, values, and attitudes that shape interactions between pupils, teachers, and administrators” (Mitchell and Bradshaw, 2013, p. 600). School climate often refers to overall interactions and relationships within the school, whereas classroom climate is considered a component of this broader construct. Classroom climate addresses “the sum of all the group processes that take place during teacher–student and student–student interactions” (Zedan, 2010, p. 76), including interpersonal relationships, classroom rules, and levels of teacher control. Perceptions of school climate may be shaped by interactions both within the classroom and across the wider school environment (Mitchell and Bradshaw, 2013). Key characteristics of school climate include a sense of safety, school belonging, the quality of student-teacher relationships, and the quality of peer relationships (Wang and Degol, 2016). Teachers are among the most significant factors in promoting a school climate grounded in care, fairness, support, and responsiveness, making teacher-student relationships particularly important (Mitchell and Bradshaw, 2013). Previous studies have found that positive, warm, and supportive teacher-student relationships are associated with students’ sense of belonging (Allen et al., 2018), school liking (Graham et al., 2022; Thornberg et al., 2023), peer relationship quality (Endedijk et al., 2022), academic engagement and achievement (Roorda et al., 2017; Thornberg et al., 2022), as well as reduced engagement in behavior problems (Lei et al., 2016) and bullying (Thornberg et al., 2024; ten Bokkel et al., 2023). Research from Sweden, where this study has been conducted, has found that more positive and supportive student-teacher relationships at the classroom level are correlated with a more positive and supportive class climate (Thornberg et al., 2023). In turn, a positive class climate is associated with reduced peer victimization (Kloo, 2025; Thornberg et al., 2024).

Mitchell and Bradshaw (2013) noted that students’ perceptions of school climate are influenced by the classroom environment, particularly the teacher’s approach to classroom management. Research also shows that students associate good teachers with qualities such as fairness, friendliness, care, respect, and positive relationships (Krane et al., 2017; Powell et al., 2018; Whitehead et al., 2023), while inconsistency, unfairness, and disrespect are linked to perceptions of poor teaching (Krane et al., 2017; Powell et al., 2018). In qualitative studies, students have also linked teacher-student relationships to their wellbeing (Newland et al., 2019; Yu et al., 2018). For example, Yu et al. (2018) found that students wanted teachers to recognize their needs inside and outside the classroom, demonstrate care, and provide help. Similarly, a recent Swedish study (Edling et al., 2025) highlighted the importance of school personnel noticing events that affect students’ wellbeing by remaining visible, intervening, and listening to students’ perspectives. Students also emphasized the need for school staff to act fairly and without bias. Thornberg et al. (2023) found that students wanted teachers to be fair and impartial not only in classroom management but also in handling student conflicts. When teachers are perceived as unfair, students may develop negative feelings and attitudes toward them (Thornberg et al., 2023), which can, in turn, affect academic achievement (Chen and Cui, 2020; Kaufman and Killen, 2022).

Unfortunately, some studies have shown that teachers may discriminate between students based on gender-related biases, both when addressing disruptive behavior and when responding to students in need of support (Myhill and Jones, 2006; Odenbring, 2019; Perander et al., 2020), including in bullying situations (Cunningham et al., 2016). One aspect of teacher-student relationships that may influence students’ academic outcomes and perceptions of their teachers is teacher bias (Kaufman and Killen, 2022; Killen et al., 2024; Thornberg et al., 2023). There is a lack of studies exploring students’ perspectives on school climate, particularly those employing qualitative methods (Konishi et al., 2022). Few studies have examined how students themselves experience and reflect upon their school climate (Bradshaw et al., 2021; Edling et al., 2025; Grazia and Molinari, 2021), and there remains a need for research investigating how students perceive gender-related aspects within school settings (Skipper and Fox, 2022; Whitehead et al., 2023).

Studies have found schools to be structured around cis- and heteronormative gender norms (Martinsson and Reimers, 2020; Pascoe, 2013), contributing to gender socialization and students’ understandings of gender that, in turn, affect the school climate and students’ subjectivities in school (Ringrose and Renold, 2010; Skipper and Fox, 2022). For example, such norms are associated with students’ engagement in gender policing, marginalization, and bullying (Bailey et al., 2022; Odenbring, 2022; Pascoe, 2013) and contribute to pushing minority students, or those perceived as defying gender and sexuality norms, to the margins (Forsberg et al., 2025a; Ringrose and Renold, 2010). These norms can also become visible in everyday routines such as classroom management and the handling of disruptive behaviors (Myhill and Jones, 2006; Odenbring, 2019; Perander et al., 2020; Skipper and Fox, 2022), as well as in how teachers intervene in student conflicts and bullying (Cunningham et al., 2016; Forsberg et al., 2025a). Some studies have shown that teachers may be influenced by gender bias (Sheehy and Solvason, 2023; Skipper and Fox, 2022), which can affect their interactions with students (Odenbring, 2019; Perander et al., 2020; Skipper and Fox, 2022) and influence how students perceive their teachers and the school environment. Studies show gender differences in learning outcomes and perceptions of students’ own capability to solve mathematical tasks (Nicchiotti and Spagnolo, 2024; Spagnolo and Nicchiotti, 2023), as well as the perception among boys that they are more closely monitored and expected to misbehave (Skipper and Fox, 2022). Other studies find no such perceived differential treatment by teachers (Skelton and Read, 2006). In the Swedish context, Odenbring (2019) demonstrated that school professionals’ discussions of students’ health-related issues were often framed by normative gender descriptions and interventions. Similarly, Keisu and Ahlström (2020) found that students reported gender issues were rarely addressed in school and that student perspectives and participation were often lacking. This is particularly noteworthy given that Swedish national policies and curricula emphasize the obligation of all school staff to promote inclusive school climates for all students, regardless of gender identity, gender expression, or sexual orientation (SFS, 2025). This responsibility also includes addressing issues related to identity, sexuality, and gender norms (Bengtsson and Bolander, 2020; Skolverket, 2023). It has been suggested that understandings of gender are fluid and that schools can “either reproduce the dominant gender ideology of the wider society or be a potential site for developing non-traditional gender identities” (Myhill and Jones, 2006, p. 100). Against this backdrop, we used a qualitative approach to explore how students perceive their school climate, focusing on student-teacher relationships and gender in the Swedish context.

## 2 Theoretical framework

### 2.1 A social-ecological framework

Bronfenbrenner’s (1977, 1979) social-ecological framework has proven valuable for understanding school climate and its various dimensions (Allen et al., 2018; Dorio et al., 2020; Forsberg et al., 2025b, Hammar Chiriac et al., 2024; Wang and Degol, 2016). Drawing on Bronfenbrenner’s model, we view the school climate as emerging from a complex interplay among individuals and four systems: microsystem, mesosystem, exosystem, and macrosystem. Collectively, these systems shape the immediate environments, such as schools, where individuals develop. When unpacking school climate through this lens, the microsystem encompasses the various social environments in which students are directly involved, such as student-teacher relationships and peer relationships. The mesosystem, in turn, refers to the interactions among these different microsystems, for example, how students’ relationships with their teachers and their relationships with peers influence one another (Bouchard and Smith, 2017). The exosystem includes contextual factors that do not directly involve the student but nonetheless influence the school climate, such as school leadership and organizational structures. Finally, the macrosystem addresses broader societal structures, ideologies, and norms, including those related to gender (Bronfenbrenner, 1977, 1979). This perspective highlights how students’ perceptions of school climate, safety, and relationships are influenced by social-ecological dimensions, including the quality of interpersonal relationships, the sense of security, and the institutional environment (Wang and Degol, 2016).

## 3 Methodology

The findings presented in this paper are part of a larger project investigating students’ and teachers’ perspectives on school climate, social climate, and student-teacher relationships (see, for example, Thornberg et al., 2022, 2023, 2024; Hammar Chiriac et al., 2024; Forsberg et al., 2025b,c). Data were collected through semi-structured focus group interviews with open-ended questions. Data collection and analysis were guided by a constructivist grounded theory (CGT) approach (Charmaz, 2014), which emphasizes participants’ primary concerns, the social processes they describe, and their interpretations of their social worlds. The study design was also influenced by the sociology of childhood, where our role as researchers can be described as guided by openness and curiosity toward students’ perspectives, approaching participants non-judgmentally, and positioning them as expert commentators during data collection (Corsaro, 2018).

### 3.1 Participants

The focus groups included students from two public schools and were conducted at two time points, 1 year apart. In total, 59 focus group interviews were conducted: 41 groups consisted of six participants, while 18 groups included 4–5 participants because of absences or illness. In the first wave, 164 students (78 boys and 86 girls) participated in 29 focus groups. In the second wave, 168 students (82 boys and 86 girls) participated in 30 focus groups. The two schools differed in geographical location, grade levels, and size. School A, located in a small village, was an elementary and lower secondary school covering grades 1–9 (ages approximately 7–15) with 410 students. School B, located in a medium-sized Swedish city, was an elementary school covering grades 1–6 (ages approximately 7–12) with 287 students. All students in grades 1–9 at School A and grades 1–6 at School B were invited to participate (*N* = 697). Regarding socio-economic dynamics, in School A, 61% of the students had highly educated parents, and 10%–12% had a foreign background (i.e., either born in another country or both parents were born in another country). In School B, 86% of the students had highly educated parents and only 5% had a foreign background.

### 3.2 Procedures

Ethical approval was obtained prior to data collection. Following approval, all students and their caregivers were informed about the project, and informed consent was obtained. Students were told that participation was voluntary, that their responses would be treated confidentially and anonymized, and that pseudonyms would be used. To enhance representativeness within the schools, the focus groups were randomly assigned within each grade and school, ensuring that all participants in a group were from the same grade, attended the same school, and identified with the same gender. During the interview session, we repeated the information about the study, emphasizing that participation was voluntary and confirming that they still wanted to take part. We explained the types of questions we would ask, that they did not have to answer any questions they were uncomfortable with, and how we viewed the students as expert commentators on their social worlds. We also stressed that we were interested in listening to what they had to say. Interview questions focused on students’ perceptions of social relationships at school, their views on teachers and student-teacher relationships, and their experiences in various school spaces, including classrooms. We probed for students’ understandings and perspectives. In the second wave, we added questions about changes or continuities compared to the previous year, informed by our analysis. Throughout the study, we prioritized participants’ wellbeing. For example, we conducted same-sex focus groups to help students feel more comfortable discussing aspects of school climate (Wallace et al., 2025; Woodring et al., 2006), a format that students tend to prefer (Strindberg, 2021). All interviews were conducted in private rooms at the students’ schools, with one interviewer present (first or third author). No signs of discomfort were observed, and students appeared engaged and interested in sharing their perspectives. Interviews lasted between 30 and 60 min and were transcribed verbatim.

### 3.3 Data analysis

In this paper, we focus our analysis on students’ perspectives on student-teacher relationships, school climate, and gender. Gender was not introduced as a topic in the interviews; rather, it emerged as a central theme during data collection and analysis, particularly in relation to student-teacher relationships. This reflects our commitment to being guided by the concerns students themselves raised. Following our grounded theory approach, we conducted initial, focused, and theoretical coding (Charmaz, 2014). All three researchers collaborated on the analysis; the second author was more involved in the later stages, whereas the first and the third author participated in all stages. Initial coding involved line-by-line and word-by-word analysis to generate conceptual codes such as injustice/justice, teasing, non-caring, fear, and inaction. The first and third author generated initial codes individually and then collaboratively refined them by comparing, discussing, writing memos, and sorting the codes. During our comparisons of codes and data, we identified a central concern: students’ perceptions of poor school climate were closely tied to student-teacher interactions involving gender dynamics. The data were coded using MAXQDA, which helped visualize, compare, cluster, and refine codes, and apply already generated codes to larger parts of the data to gradually shift from initial to focused coding. In the focused coding phase, we used the most recurring codes to explore students’ central concerns in greater detail across the dataset and asked analytical questions such as under what circumstances gender dynamics were raised and with which codes these dynamics were associated. In this stage, codes were developed into categories. For example, codes addressing issues such as injustice/justice and unfairness during teacher-led situations were conceptualized as *unfair differentiating*. In total, we conceptualized three categories: *unfair differentiating*, *fragmented interventions*, and *gender reinforcing*. Overall, our findings show that students questioned and problematized certain interactions they experienced as biased, unfair, or lacking in interventions. To conceptualize these experiences, during theoretical coding we developed the social process of *Distrustful Encounters*, as the three categories together illustrated encounters that students viewed as problematic, with attention to gender dynamics. *Distrustful Encounters* illustrates how students interpret situations and interactions and how these shaped their perceptions of student-teacher relationships in relation to gender and school climate. These were the examples and patterns of distrustful encounters that students raised, exemplified, and discussed during the focus group interviews, and that we found possible to elaborate on during data collection and our analysis. To further theoretically understand students’ perspectives on student-teacher relationships, gender, and school climate, we incorporated a social-ecological framework.

### 3.4 Researcher reflexivity

As a research team, we recognize that our positionalities, shaped by our gender identities and professional backgrounds, inform both our engagement with participants and our co-construction and interpretation of data. The team comprises two female researchers and one male researcher. We remained reflexive throughout the research process, critically discussing how our assumptions, disciplinary lenses, and positionalities might influence data gathering, coding, and analysis. The research group is heterogeneous in terms of gender, age, and subject affiliation, and all members have extensive experience interacting with children in school-based research contexts and collecting data through focus groups with children. Two of us are leading researchers in CGT, which ensures that the theoretical framework and coding decisions are highly credible and grounded in theoretical knowledge and expertise. Our collaborative approach to analyzing the empirical material and interpreting the results further strengthens the quality and validity of the analysis.

## 4 Results

From the students’ narratives, a social process termed *distrustful encounters* was conceptualized. This process refers to experiences of being treated unfairly, receiving non-caring or fragmented interventions when care was needed, and encountering gender-reinforcing behaviors. According to the students, these experiences led to feelings of neglect and unfair treatment. While there were some differences in how girls and boys experienced these interactions, both groups described instances of unfair differentiating, fragmented interventions, and gender reinforcing. These interactions occurred not only in classroom settings but also in other school contexts, such as playgrounds and corridors during recess. At times, students questioned these interactions and managed to navigate or alter the situation. However, in other instances, they found it more difficult to speak up, either because they feared consequences or their concerns were not acknowledged.

While this paper focuses on distrustful encounters, it is important to note that students also described trustful student-teacher relationships (see, for example, Thornberg et al., 2023; Forsberg et al., 2025b,c). However, when examining their experiences of negative school climate through a gender lens, distrustful encounters emerged as a central concern, particularly in relation to interactions that students perceived as problematic or unjust in their daily relationships with teachers. From a social-ecological perspective, the microsystem, specifically the quality of student-teacher relationships, was crucial in shaping students’ experiences of school climate. As will be demonstrated throughout this paper, macrosystem-level gender norms appeared to influence the characteristics of these relationships.

Thus, rather than attributing blame to individual teachers, the gendered dimensions manifested in these social interactions can be understood in relation to broader societal and cultural gender norms and expectations (Martinsson and Reimers, 2020; Pascoe, 2013). While in some cases this influence was associated with individual teachers, more often broader patterns emerged, indicating that groups of teachers across various school contexts engaged in these types of interactions. Although students at both schools reported distrustful encounters, there was a slight notable difference between the two. Students at School A gave more examples of distrustful encounters compared to those at School B. This pattern seemed to particularly affect boys at School A, who provided more examples of distrustful encounters.

This may suggest that taken-for-granted macrosystem-level norms exerted a stronger influence across the exo-, meso-, and microsystems at School A.

### 4.1 Unfair differentiating

A recurring theme in the students’ narratives about teacher-student interactions was what we conceptualize as *unfair differentiating*. Students frequently expressed a desire for fairness in their interactions with teachers, emphasizing that fairness was a key characteristic of a good teacher.

Interviewer: How do you think a good school should be?
John: Equal opportunities
Mark: Yes, equal opportunities
John: That no teacher shows favoritism.
Mark: Exactly.
John: Like not just listening to the girls or just listening to the boys but both (Boys, grade 6, School B).

As illustrated in this example, a good school was associated with fair and unbiased teachers, those who listened to all students, avoided scolding the wrong student, and responded to behaviors consistently and equitably. Many of the students’ examples of teachers perceived as engaging in unfair differentiating involved situations where boys and girls were treated differently. For instance, boys often reported feeling that they were more frequently reprimanded or scolded for disruptive behavior and conflicts. This was reflected by Jeff and Peter in this example:

Jeff: I think it’s so strange that they only tell us boys off, but they don’t say anything to the girls when something happens. Like if boys get into a fight with Carine and - then they tell us off, but if girls get into a fight, they don’t say anything. And the same thing if girls are late, very late, if we are like five minutes late, they tell us off a lot, so we have to stay behind, but girls, if they are ten minutes late, they don’t have to stay behind at all.
Peter: I remember that Carine was really late.
Jeff: She misses the first lesson every day (Boys, grade 5, School A).

According to the boys in this example, the unfair scolding was a recurring experience, often contrasted with situations where girls were not reprimanded for similar behaviors. This perceived unfairness also extended to other consequences, such as being required to stay behind after class, highlighting inconsistencies in teachers’ practices. From the girls’ perspective, they acknowledged that they were not scolded, even though, as they pointed out, girls could behave just as poorly.

But it’s silly that teachers seem to have such a hard time telling off girls, but they find it very easy to tell off boys. I get very upset about it because I deserve more scolding than I get. If I was a boy and did all those things, if I - uh, like the things I do, uh, then I would get like a life sentence, no (laughs) but now that I’m a girl, it’s - or well, maybe they’re not as used to girls acting up, but I’m a bit rebellious and very -. But all the girls in the class except - or all the girls in the class except me aren’t as annoying as some boys, I think (Angel, grade, 6, School A).

In the excerpt above, Angel reflects on how unfair differentiating practices occur in the classroom and how she can get away with behaviors for which boys would typically be reprimanded. Angel also raises the issue of gender norms and teachers’ perceptions, which may contribute to this unequal treatment in classroom management. Angel’s behavior is perceived as an exception, while boys as a group are positioned as more responsible for disruptive or annoying behavior. In this way, Angel may also be drawing on and benefiting from prevailing gender norms. Girls also shared experiences of unfair differentiating, especially in relation to physical education classes. One example concerned how the PE teacher acted during class. In these instances, some girls felt they were treated unfairly compared to boys, as illustrated in the example below.

Maria: And then if they ask all the students—so it’s not just a few they usually ask—it becomes fairer for everyone. But/…/the teachers say, “Ah, what do you think we should do here?” like in PE, and then it’s usually the boys who answer, and we girls don’t get a say. Then it becomes—we always do what they want
Sonya: Yes, someone shouts floorball and then–, and then everyone plays floorball because no one dares to speak up, because then you get a comment. So, a good school would be like the teacher really makes sure everyone gets to say what they think, and if you hear comments, it’s like–, it should be unacceptable to make comments (Girls, Grade 9, School A).

As illustrated in this example, the manner in which the teacher involves students in decision-making during PE classes tends to privilege boys’ suggestions. The girls propose alternative approaches to structuring these interactions to challenge such unfair practices. They also highlight the difficulties students may encounter when attempting to speak up about unfair differentiating practices. As noted here, there is a persistent risk of receiving negative comments from boys, which can discourage girls from voicing their concerns due to fear of reprisal. Furthermore, the students emphasize the need for teachers to respond to such comments, suggesting that there has been a lack of intervention in similar situations in the past. The fear of negative consequences was also reported by some boys, who shared that they did not dare to speak up about teachers’ unfair differentiating in the classroom because they were afraid of being reprimanded for doing so.

John: Teacher, can’t you hear those sitting at the back?
Interviewer: Yes. Did you say that?
John: No.
Mark: No, because you don’t dare to say it.
John: No, you don’t dare to stand in the middle of the classroom and.
Interviewer: Why don’t you dare to say it, what are you afraid of?
Mark: That you’ll get scolded more. Standing in the middle by the teacher’s desk and just. now it’s the one who talks the most with. that letter in the last name (Boys, grade 6, School B).

As discussed by the boys, they feared speaking up about girls’ disruptive behavior because they believed it would backfire, including the risk of public humiliation. Boys therefore appeared to fear consequences from teachers if they protested against unfair differentiating in the classroom, while girls feared negative reactions from boys if they questioned unfair practices during PE.

Overall, this section highlights how both girls and boys identified and problematized unfair differentiation. While boys more frequently described experiences of being unfairly scolded and punished in relation to classroom management, girls pointed to examples from PE lessons. These accounts illustrate how students perceived gender-based unfair treatment in two distinct school settings, both of which are traditionally gendered spaces. The PE setting in particular has been associated with hegemonic masculinity norms that affect girls’ experiences as well as those of students with non-dominant masculinities or gender expressions (Herrick and Duncan, 2023; Hortigüela-Alcalá et al., 2021). Similarly, the association of boys with trouble and their more frequent reprimanding has been well documented (Odenbring, 2019; Skipper and Fox, 2022).

These examples further underscore how gender norms shape teaching practices, not only in managing classroom behavior but also in structuring PE lessons. Students problematized these interactions as inappropriate and unfair, yet found it difficult to voice their concerns. For them, these practices were linked to a lack of trust: boys feared that teachers would not respond positively if they spoke up, while girls associated good teaching with the ability to organize PE in ways that included their voices, implying that failure to do so reflected poor teaching practices. At the microsystem level, the quality of student-teacher relationships, particularly in relation to classroom management, emerged as a critical factor. The next section addresses the second category, which focuses on intervention practices in response to student conflicts or violence and how these interventions (or lack thereof) were perceived through a gendered lens.

### 4.2 Fragmented interventions

The second category, conceptualized from the students’ narratives, captures interactions in which students expressed a need for teacher intervention but often experienced passivity or a lack of response, thereby showing how interventions could be fragmented. From the students’ perspective, interventions were particularly necessary in situations involving peer-to-peer conflicts or more serious incidents of violence. However, students sometimes felt that teachers remained passive in these situations and failed to intervene when support was needed.

Examples mainly occurred during recess and involved violent or harassing situations between students, with boys in particular described as engaging in these episodes.

Billy: He attacked her because she asked him if he could be quiet. He pushed her head down like this into the floor. And then he strangled a boy on the ramp because he kind of told him to move since he was standing right in the middle of it.
Interviewer: What happened during these incidents? What happened?
Billy: All the teachers were kind of scared of him. It’s like with Benji, the chubby
Interviewer: But what did the teachers do then?
Billy: Kind of nothing.
Interviewer: He could go on strangling her and hitting, and no one came and did anything?
Billy: Well, they did something, but not right away… and he kind of started hitting them, and then they became pussies and walked away (Boys grade 7, School A).

These violent episodes were described as occurring in close proximity to teachers and appeared to happen repeatedly. In relation to these incidents, teachers were portrayed as passive, failing to intervene, and in some cases even being mocked, such as being called “pussies” for appearing afraid of a violent boy and walking away from the situation. In this example, the teachers themselves also seemed to be targets of aggression. By using the term “pussies,” Billy employed gender-normative language to position the teachers. Some boys from School A even expressed that it was their peers, rather than teachers, who stepped in during violent situations. This frustration is captured in Tony’s account: “A freaking teacher there and just watches, she doesn’t do anything, she just stands there. So then my other friend has to push him away. But the teacher just stands there and just watches, she doesn’t do anything” (Tony, Grade 7, School A). The incidents described can be understood as manifestations of gendered practices that unfold during unstructured times, such as recess, where boys are often expected to behave aggressively during games. Similar gender dynamics have been observed in relation to the football pitch (Forsberg et al., 2023) and other school spaces (Bhana and Mayeza, 2016; Mayeza and Bhana, 2020). As noted earlier, the lack of intervention was sometimes attributed to teachers being perceived as afraid. If teachers themselves were harassed or attacked, this may have influenced their willingness or ability to intervene. While violent incidents involving non-intervention were primarily reported by students at School A, similar interactions had also occurred previously at School B.

Lukas: Yeah, in fourth grade, we had a different mentor who wasn’t as… didn’t tell us off as much, and there was this one lesson when someone had been punched in the stomach, like, the whole lesson for about an hour, just lying on the ground. But that doesn’t happen now that we have other teachers who can step in.
Mattias: It was just straight up… that teacher didn’t dare to say anything, because he was new then, so… they took the opportunity to cause more trouble, because they knew no one would tell them off (Boys, Grade 6, School B).

As described in this excerpt, a violent episode occurred in front of a teacher in the classroom, but the teacher did not dare to intervene. The lack of intervention was attributed to the teacher being new, and this was contrasted with how the situation had improved over time, with teachers now showing care and taking action. At School A, there was also an example of a teacher intervention where a male teacher was described almost like a superhero for taking strong and decisive action. The girls in this example expressed disbelief, saying they could hardly believe their eyes when the teacher stepped in during a violent incident.

Interviewer: But if something happens, do the teachers come over and help?
Patricia: No, not that often.
Sandra: We had our PE teacher, our former PE teacher, he, what’s it called, there was a fight, and then, like, he really, he was super fast, so he really ran, he did everything to stop the fight.
Tania: He threw off his jacket, he threw off his backpack really fast, and then he ran after them.
Mandy: That was to stop the fight.
Laura: You couldn’t believe your eyes. He was super-fast (Girls, grade 4, School A).

According to these girls, most teachers were perceived as passive and non-intervening, whereas the male PE teacher was described as taking action and intervening in a superhero-like manner to stop the fight. The intervention described here reflects a normative response to violent episodes where incidents showing patterns of hegemonic masculinity among students are met with interventions following a similar gender dynamic. These incidents often involved boys, girls were also present as observers and, at times, as targets. Some girls described situations in which boys pushed them or used derogatory language, such as calling them “pussies,” without any significant response from teachers.

*Nina*: I also played floorball, I was like the goalkeeper, yeah, and then, when I was the goalkeeper, the ball came and I got scared and moved a bit to the side, and then Olof came and just pushed me and said, “Oh, are you a coward or what?” and just started pushing me for no reason. Yeah, maybe I am a bit of a coward, so what?
*Interviewer*: What happens then when you tell someone? Because it doesn’t sound like—are there any teachers around when these things happen?
*Nina*: Yeah, there are some teachers around.
*Bella*: Not that many…
*Nina*: There are yard duty teachers, but we’ve talked about how they also just stand in a group and talk, so we’ve said in student council, or, well, you have to bring it up in class council first, we’ve said in class council that they need to spread out more to keep an eye on larger areas and what’s going on. And now maybe there needs to be like one teacher by each rink, or like, there needs to be some standing around.
*Interviewer*: Right.
*Nina*: So they can really step in if something happens.
*Bella*: Yeah.
*Nina*: But I don’t feel like it’s helped that much that we brought it up in student council. They still just stand in a group and talk. I could hit Vanessa right next to them (Girls, grade 3, School A).

While fear was again mentioned as one reason for the lack of teacher interventions, students also linked this inaction to inadequate supervision practices, as highlighted above. The incident also illustrates how students themselves engage in cis- and heteronormative practices, where the boy in the incident acts aggressively and calls the girl a coward (Bhana and Mayeza, 2016; Mayeza and Bhana, 2020). While students had tried to change the situation by formally addressing the issue during class and school advisory board meetings, no improvements had yet been made. According to the students, insufficient supervision contributed to these incidents occurring and to the lack of timely intervention. This issue may be influenced by exosystem-level organizational factors, such as the number of school personnel and the size of outdoor spaces, both of which have been shown to affect teacher supervision (Horton et al., 2020). Additionally, how schools address gendered dimensions in areas like sports and play spaces may also play a role (Forsberg et al., 2023). In contrast, students also talked about how some teachers had intervened to stop boys from harassing girls during recess.

Yeah, one teacher has really brought it up a lot with those boys. So I think… he even missed a whole lesson because he just sat and talked with that student. And then you know that… and now they’ve mostly stopped, so you can tell he really dealt with it (Frida, grade 5, School B).

As highlighted by Frida, the teacher was perceived as helpful for taking the time to speak with the boys, which in turn had a positive impact on the situation. This example also illustrates how interventions varied, ranging from primarily no intervention in violent and harassing incidents to normative actions and measures that improved the situation.

Overall, this section shows that while students desire teacher interventions, such interventions do not always occur, do not happen in the moment, or fail to prevent similar incidents from recurring, even after the issue has been addressed. This suggests that interventions are often fragmented, either due to supervision practices that allow gendered disputes or harassment from boys to go unnoticed or due to a lack of response during violent episodes, particularly those involving boys. Some boys even shared examples where peers, rather than teachers, intervened, leaving them feeling isolated in managing the situation. These accounts may indicate that violence has become normalized or widespread (Borg, 2023). Contributing factors may include exosystem-level conditions such as limited resources, the size of school grounds, and the presence (or absence) of gender-sensitive organizational practices (Borg, 2023; Forsberg et al., 2025a). School A was a larger school with more students, including older students, compared to School B. The two schools were also located in different neighborhoods with slightly different socio-economic dynamics (see section “3 Methodology”). These factors may help explain the differences observed between the two schools. At School A, students described more consistent patterns of non-intervention and violent episodes, whereas at School B, students reported changes in these dynamics and more frequent teacher interventions. On an exosystem level, time, resources, and organizational planning must be allocated to ensure that teachers are better equipped to intervene effectively. This is especially important, as fragmented interventions negatively affect students’ perceptions of their relationships with teachers. At this point, particularly in School A, such patterns may even contribute to the reproduction of dominant gender norms (Myhill and Jones, 2006). This was also evident in the third category, where students at School A provided examples of various gender-reinforcing interactions.

### 4.3 Gender reinforcing

The third type of distrustful encounter, conceptualized from the students’ narratives, was reported exclusively by students at School A, suggesting that the nature of interactions and, consequently, the school climate may have differed between the two schools. These interactions highlight instances of gender-reinforcing behavior by some teachers, visible in a number of situations. One example frequently mentioned by students was the football challenge arranged between boys and male adults, most of whom were teachers. This example illustrates gender reinforcement, in which adults themselves reinforce gender-normative behaviors.

*Billy*: And then we were going to play a football against them. The teachers.
*Jonas*: Oh right.
*Billy*: And they just ran all over us.
*Fredrik*: The sixth graders always play against the teachers.
*Billy*: The ones who worked at the daycare, or the after-school club, they took it so damn seriously.
*Interviewer*: And you didn’t?
*Billy*: No, I mean, it’s pretty hard to try to beat 30-year-olds. They also played in their free time.
*Fredrik*: Yeah, they did. Half of them.
*Rasmus*: Yeah, we also had one of those damn matches, and our school was being renovated at the time. Then it got unfair, because the construction workers joined in, and one of them had played serious football at a high level. And they played completely seriously, so it was impossible to win.
*Billy*: My teacher Harry, though, he tried so damn hard, he stamped on me and pushed me.
*Rasmus*: Yeah, seriously, what the hell (Boys, grade 7, School A).

According to the boys, the adults, due to their age and football skills, took the challenge very seriously. As the boys explained, this football challenge was a regular activity at the school, suggesting that this gendered practice was institutionalized at the exosystem level. The event seemed to leave the boys feeling overpowered, with teams perceived as unequally matched. This institutionalized gender practice, in which adults themselves enforced normative gender behaviors, involved a form of heteronormative masculinity challenge where male teachers and construction workers, aware of their superior age and football skills, approached the game with great seriousness. As a result, the boys felt they were unable to participate on equal terms. One boy also described how his own teacher engaged in rough play, which the boys perceived as excessive, reinforcing the sense of being “put in their place” during the challenge. Previous research has shown that rough play on the school football pitch can be a difficult gendered arena, where hegemonic masculinity and exclusionary practices are common (Martínez-Andrés et al., 2017; Forsberg et al., 2023). Engaging in such heteronormative masculinity challenges may therefore be counterproductive if the goal is to address these dynamics among students, particularly among boys, and may instead reinforce cis- and heteronormative gender norms both on the football pitch and within the broader school climate.

Another set of interactions that exemplified gender reinforcement related to recess and situations in which students might need support, but instead described instances where teachers responded in a gender-stereotypical way, providing non-care and being dismissive of boys in need of care.

*Andreas*: But when Lars got hurt he was really mean afterward. And then when Noah got hurt, he didn’t care at all, he just, he didn’t care, so he didn’t even go and ask how he was.
*Interviewer*: How did that feel?
*Noah*: Yeah, bad. It was like when I was doing high jump, I jumped like one twenty and slid off the mat, and I hit my back because I slid off and landed on the floor, and he just said, “Get up now, it’s nothing.”
*Andreas*: He’s also really mean, because—
*Björn*: It feels like he shouldn’t be working at this school, I think (Boys, grade 2, School A).

These boys perceived the teacher as uncaring and uninterested in helping, even deeming him unsuitable to work in a school because of his actions. This reflects a student–teacher relationship lacking trust and care. These examples of distrustful encounters, along with the expression “get up now, it’s nothing,” where care is expected, suggest a practice of gender policing demanding the performance of intelligible genders (Ringrose and Renold, 2010; Payne and Smith, 2022; Rawlings, 2017). Such gender policing also takes place within a cis-heteronormative order, which was evident in an example raised by some of the girls. In this example, the girls discussed how they would not seek support from certain adults at school due to a lack of trust, based on previous interactions where some of these adults had teased one of the girls for playing with a boy.

Lilly: There are quite a few yard duty teachers, but I usually don’t want to go to the ones who work in after-school care because they tend to be a bit more teasing. For example, one of them teased me: I was with a boy and he said, “Are you two together?” and stuff like that. We said no, but he kept saying we were together anyway, and…
Felicia: And that’s an adult.
Interviewer: Who works in after-school care?
Lilly: So we told our homeroom teacher, who took care of it and talked to the principal and so on, saying that he needed to behave better toward the kids (Girls, grade 5, School A).

According to the girls, these types of interactions also revealed a lack of trust in their relationships with certain teachers. Rather than feeling supported, such interactions led them to avoid turning to those teachers when they needed help. The teasing itself can be seen as an example of the teacher reinforcing gender-normative behaviors, reflecting a cis-heteronormative logic, particularly when friendships between girls and boys were ridiculed by the teacher. In contrast to other situations where students found it difficult to speak up about problematic interactions, the girls in this example indicated that they had trustful relationships with other teachers. These trusted teachers were seen as allies who could help address and prevent such interactions from recurring. This highlights how some microsystem-level relationships were built on trust and care, and how a homeroom teacher, for instance, could influence change within the mesosystem by addressing issues among colleagues. However, taken together, the examples discussed by the students seem to suggest that the school was structured around cis- and heteronormative gender norms (Martinsson and Reimers, 2020; Pascoe, 2013) that appeared institutionalized at the exosystem level. A final example, taking place between boys and one of their female teachers, illustrates a verbal comment showing a gender-stereotypical positioning of boys as a group as less worthy or less mature.

*Mårten*:/…/Britta says that girls are worth more than boys.
*Paul*: Yeah.
*Ronny*: A lot of teachers at the school say that—
*Benjamin*: No.
*Ronny*: —say that girls are worth more.
*Magnus*: Yeah, because they give birth.
*Mårten*: They’re like, “Girls are better, they mature faster, they’re worth more.”
*Paul*: Yeah, Britta actually said that (Boys grade 5, School A).

In the boys’ discussions, several statements revealed how girls, as a group, were spoken of as being valued more highly than boys. These statements reflect a gender-reinforcing approach rooted in cis- and heteronormative norms. While one of the boys appeared to object to such interactions, experiences of unfair differentiation in the classroom, lack of teacher intervention, and other gender-reinforcing behaviors may negatively influence how boys perceive their student–teacher relationships and the overall school climate. Compared to girls, boys at times seemed to have fewer options for addressing these issues. Some expressed that many teachers engaged in such behaviors, making it difficult to find support or influence change within the teacher-to-teacher mesosystem. As highlighted by Edling et al. (2025), boys may be more “trapped in negative situations without more viable options” (p. 13). What becomes evident in this section, however, is that both boys and girls question and problematize gender-reinforcing interactions based on cis- and heteronormative norms that seem institutionalized at the exosystem level at the school, appearing in a variety of interactions and situations between teachers and students. Students call for alternative practices, as these experiences lead them to feel that their wellbeing is not prioritized and that their trust in certain teachers has been broken.

## 5 Discussion and limitations

### 5.1 Discussion

This study set out to explore students’ perspectives on school climate, student–teacher relationships, and gender. As shown in previous research, the association between a positive school climate and the quality of student–teacher relationships is crucial (Endedijk et al., 2022; Thornberg et al., 2022, 2023; Wang and Degol, 2016), influencing learning, safety, and engagement in conflict. Students tend to perceive their school climate more positively when teachers are fair, caring, and responsive to student conflicts and bullying (Thornberg et al., 2023). This underscores the importance of student–teacher relationships for both academic outcomes (Roorda et al., 2017) and students’ social wellbeing, peer relationships, and safety (Endedijk et al., 2022; Kloo et al., 2023; Thornberg et al., 2023; Forsberg et al., 2025b). From a social-ecological perspective (Bronfenbrenner, 1977, 1979), the student–teacher relationship (microsystem) plays a central role in shaping students’ perceptions of their teachers and the broader school climate.

Our findings suggest that when students experienced distrustful encounters, they felt unfairly treated or uncared for. For boys, such encounters occurred in classrooms, during recess, and in other interactions with teachers, particularly at School A. This may indicate broader mesosystem-level issues affecting boys’ immediate interactions with various teachers. Additionally, exosystem factors such as how supervision is organized may contribute to these experiences, particularly in cases of non-intervention. Boys at School B also noted differences in teacher interventions, suggesting that longer-term relationships with teachers could lead to shifts in the broader teacher group and more consistent interventions. For girls, sport-related contexts, whether during PE or informal games at recess, were situations where they felt a lack of intervention or a lack of thoughtful approaches to ensure they were heard and cared for. Despite efforts to address these issues through the student council, the girls were unable to influence the exosystem. However, they sometimes succeeded in receiving support from trusted teachers, which in turn helped influence other teachers and improve student–teacher relationships.

Cis- and heteronormative gender norms at the macrosystem level appeared to influence school climate and student–teacher interactions at both schools, though more prominently at School A. Students at School A reported gender-reinforcing interactions that were not mentioned by students at School B. This may suggest that School A, at the time of the study, was reproducing more dominant gender patterns across the micro-, meso-, and exosystem levels (Myhill and Jones, 2006) and that these norms might be institutionalized at the meso- and exosystem levels, illustrated in a number of situations and interactions at the microsystem level. It is also important to note that School A reported more violent episodes, including some directed at teachers. From a social-ecological perspective, these microsystem-level interactions may have influenced the meso- and exosystem levels in a complex interplay.

Noteworthy is that students engaged in cis- and heteronormative practices themselves; for example, girls reported incidents where boys had harassed them, and several violent incidents among boys were reported. This might suggest differences in school climate between the two schools that shaped students’ perceptions and experiences. From a social-ecological perspective, such differences might relate to mesosystem factors such as the neighborhood in which the school was located, exosystem factors such as the size of the school, or microsystem factors such as group characteristics, together with the influence of macrosystem norms. While there are some variations between the schools in terms of socio-economic and geographical locations, we do not have sufficient data to support how such factors influence students’ perspectives on their school climate. However, based on earlier research, variations between schools are connected to school climate, and research on school climate has utilized a social-ecological framework suggesting such factors are important (Allen et al., 2018; Dorio et al., 2020; Thornberg et al., 2023; Wang and Degol, 2016). Future research should employ analyses in which such dimensions can be captured, as this might provide tools for practical implications.

Research on students’ perspectives on school climate remains limited, especially when incorporating a gender lens. In our study, students described what we conceptualized as distrustful encounters—interactions shaped by gender and gender norms. These encounters emerged organically as students reflected on their broader experiences of school climate and relationships. In contrast to positive, warm, fair, and caring relationships, these encounters revealed moments of distrust, where students felt unfairly treated, uncared for, or devalued as a group. The consequences of these encounters, as described by students, included feelings of powerlessness, particularly in relation to unfair differentiation, being left to handle conflicts alone, and frustration over interactions that were difficult to challenge. Some students reported that they felt certain teachers were unsuitable and should not work in schools. However, students also described moments of change, where patterns shifted due to stronger student–teacher relationships or support from other trusted teachers. These narratives show that students actively question and problematize interactions they perceive as unfair, passive bystanding, or gender-reinforcing. In doing so, they also articulate their vision for more supportive student–teacher relationships and a more inclusive school climate. This highlights the need for positive, caring, fair, and trustful relationships free from bias and unfair treatment. The long-term consequences of experiencing relationships as distrustful may, as previous research suggests, impact learning, achievement, social dynamics, and safety (Aldridge and McChesney, 2018; Graham et al., 2022; Kloo, 2025; Roorda et al., 2017). Not being cared for or being left to manage violence alone may leave students feeling isolated. Similarly, being excluded from shaping PE lessons may affect students’ motivation and sense of being heard. The students’ narratives call for a more gender-aware approach (Bengtsson and Bolander, 2020). It is also worth noting that while girls sometimes acknowledged the distrustful encounters boys experienced, boys did not highlight the experiences of girls. The types of gender-reinforcing interactions described in our study are particularly problematic in light of the responsibility Swedish schools have to provide inclusive school climates for all students, regardless of gender identity, gender expression, or sexual orientation (SFS, 2025), and to address issues related to identity, sexuality, and gender norms (Bengtsson and Bolander, 2020; Skolverket, 2023). Furthermore, the students’ narratives followed a binary understanding of gender, focusing only on girls and boys. However, since the topic of gender was raised by the students themselves and not introduced by the researchers, these examples likely reflect their primary concerns within a cisgender and heteronormative context. That said, students did problematize stereotypical interactions and heteronormative teasing, indicating a critical awareness of these dynamics. In constructivist grounded theory, data are viewed as co-constructed and the theory as modifiable; by conducting, for example, individual interviews, other examples of distrustful encounters might have been raised with attention to marginalized youth students’ experiences. However, such perspectives might also be difficult to raise if the school context is dominated by cis- and heteronormativity. As our findings illustrate, and as previously shown in a number of studies, the institutional context of schooling is shaped by cis- and heteronormative norms (Martinsson and Reimers, 2020; Martino et al., 2022; Payne and Smith, 2022), affecting both how issues related to gender are engaged with and tendencies to favor cis- and heteronormative subjectivities (Ringrose and Renold, 2010) as well as illustrated in students’ own peer-to-peer interactions. Some practical implications for how to move away from such patterns and instead promote an inclusive and positive school climate will have to include systematic efforts, particularly at the meso- and exosystem levels. For example, there is a need to work toward a whole-school approach where aspects such as positive, warm, and supportive teacher–student relationships are promoted throughout the organization. Focusing on the quality of the student–teacher relationship in the classroom and at the school level has a crucial impact on both social and academic outcomes. At the exosystem level, it seems essential to allocate time, resources, and organizational planning to ensure that teachers are better equipped to intervene effectively. This is crucial, as we acknowledge that there might be several barriers associated with this work, such as access to staff, finances, the size of the schoolyard, the number of interactions to manage, and the risk of being exposed to violence (Horton et al., 2020; Borg, 2023). In addition, raising awareness around gender dimensions, enforcing laws and curriculum, and, importantly, listening to student voices and including students’ perspectives in the work toward a more positive school climate are essential.

### 5.2 Limitations

This study comes with some limitations. The data were gathered from only two public schools, which may limit the generalizability of the results to other educational contexts, particularly those with different demographic or socio-economic profiles. Both schools had relatively high proportions of students with highly educated parents and a low proportion of students with a foreign background, especially School B, which may not reflect the diversity present in other Swedish schools or international contexts. While random assignment within grades and gender was used to form the focus groups, participation was voluntary, and some groups were smaller due to absences or illness. This could introduce selection bias, as students who chose to participate or were present on the day of the interview may differ systematically from those who did not participate. It might also affect the group dynamics in several ways, such as the perspectives and narratives raised during the interviews, because the groups were not based, for example, on existing friendship groups or self-selection.

The reliance on self-reported data from focus group interviews means that findings are based on students’ perceptions and recollections, which may be influenced by social desirability, bias, or group dynamics. The presence of peers in group interviews can both encourage and inhibit disclosure, potentially affecting the richness and candor of the data. Moreover, these narratives might not fully reflect what is happening in their everyday, real-life setting, and our findings might not be representative of the wider school context or other contexts. Ecological validity might be strengthened in future research by using fieldwork data, individual interviews, and video observations. In addition, we conducted gender-segregated focus group interviews as students usually form same-gender friendships and might feel safer in such a setting to talk about more sensitive issues. This might, however, have affected nuances, introduced bias in reporting, and shifted focus away from other social processes such as minority youth perspectives. However, given the examples provided regarding gender and school climate, these examples might not have been raised in a more mixed-group setting either. In this paper, we also focused on student–teacher relationships, school climate, and gender. In some of our other published papers, students have also talked about what works and provided more positive examples, not in relation to gender but more generally when discussing student–teacher relationships. Gender dynamics were thus raised as a concern when talking about what does not work. In this paper we raise students narratives on school climate and their examples on these matters.

## Data Availability

The datasets presented in this article are not readily available due to ethical considerations and data protection legislation. Requests to access the datasets should be directed to CF, camilla.forsberg@liu.se.
